# Cone-beam computed tomography for assessment of dens invaginatus in the Polish population

**DOI:** 10.1007/s11282-017-0295-7

**Published:** 2017-06-24

**Authors:** T. Katarzyna Różyło, Ingrid Różyło-Kalinowska, Magdalena Piskórz

**Affiliations:** 1 Department of Dental and Maxillofacial Radiology, Medical University of Lublin, ul. Karmelicka 7 (Stomatologiczne Centrum Kliniczne), 20-081 Lublin, Poland; 2 Independent Unit of Propedeutics of Dental and Maxillofacial Radiology, Medical University of Lublin, ul. Karmelicka 7, 20-081 Lublin, Poland

**Keywords:** Dens invaginatus, Dens in dente, Cone-beam computed tomography, Developmental variation, Radiological examination

## Abstract

**Objectives:**

There are many developmental variations in the permanent dentition. Dens invaginatus can be recognized on many dental X-rays of affected patients, but not every image allows for assessment of the type of malformation. The aim of the present study was to assess the presence of dens invaginatus with radiological features using cone-beam computed tomography (CBCT).

**Methods:**

CBCT images of 33 patients were evaluated. Age, sex, side, lateralization, occurrence in a particular group of teeth, type of invagination, differentiation, and the consequences of these factors were analyzed.

**Results:**

Forty-one teeth with dens invaginatus met the inclusion criteria for this evaluation. Females were affected more frequently than males (57.6 vs. 42.4%, respectively). The patients’ age ranged from 7 to 40 years, and the occurrence of dens invaginatus peaked from age 9 to 13 years. In total, 92.7% of affected teeth were present in the maxilla, more often unilaterally (75.8%) than bilaterally (24.2%). The most frequent tooth with dens invaginatus was the maxillary lateral incisor (53.7% of affected teeth). Almost two-thirds (63.4%) of affected teeth were found on the left side and 36.6% were found on the right. The tooth anatomy was distorted within the crown and root. Dens invaginatus sometimes affected other surrounding teeth and reduced their esthetics.

**Conclusions:**

The obtained data indicate that CBCT examination is an essential tool in assessing dens invaginatus and can guide dental practitioners in treating patients who exhibit characteristic features of this disorder. CBCT allows the clinician to distinguish the type of anomaly.

## Introduction

Dens invaginatus (DI), also known as dens in dente, is a dental malformation found mostly in the permanent dentition. The etiopathogenesis is unclear. Various theories concerning the formation of this anomaly have been proposed; however, according to the most widely accepted theory, DI is caused by invagination of the enamel into the adjacent dental papilla during tooth development [1]. The most common type of DI involves invagination of the crown, but DI can also involve the root or both the crown and the root. With regard to the severity of the anomaly, either small invaginations in the crown accompanied by a reduction in the pulp chamber volume may be observed (foramen cecum), or very deep invaginations including the root of the affected tooth may occur, resulting in a characteristic radiological image called a “tooth within a tooth”. The radiological image is very specific, because the enamel forms layers of opacities inside the dentin, which can have a linear shape. The crown of the invaginated tooth can be normal or peg-shaped. Food debris is difficult to clean from the groove in the area of the invagination, promoting caries development; as a consequence, an incorrect connection between the oral cavity and pulp chamber may lead to the development of periapical lesions. Root invagination presents as an ill-defined radiolucency along the root. Huge anomalies in the form of DI cause considerable tooth malformation [2].

DI can be recognized on almost all types of dental X-rays, but the advantage of cone-beam computed tomography (CBCT) examination is assessment of the type of malformation. The following widely accepted classification of DI was proposed by Oehlers [3, 4]:

Type 1: Enamel-lined invagination confined to the coronal part of the tooth
Type 2: Extension of the invagination into the root, beyond the cementoenamel junction, ending as a blind sac; the invagination may communicate with the pulp
Type 3a: Includes permeation of the root by the invagination, forming an additional lateral foramen; usually, there is no communication with the pulp
Type 3b: Includes permeation of the root by the invagination, forming an additional apical foramen; usually, there is no communication with the pulp

A more detailed classification was proposed in 1972 by Schulze and Brand. This classification includes 12 variations of DI, with the invaginations starting from the incisal edge or the top of the crown, and describes dysmorphic root configurations [3]. The classification is divided into groups a and b, with four different variations in each group. In the first group, the invagination causes division of the enamel and dentin. In the second group, the invagination causes division of the pulp chamber into two, along with division of the enamel and dentin [5].

The use of CBCT should always be justified by an analysis and optimization of the risk/benefit ratio for a given patient, because it involves higher radiation exposure than that associated with periapical radiography [6]. CBCT examination also has other limitations; for example, because DI mostly affects adolescent patients (age of 7–13 years), lack of cooperation during the scan can be a limiting factor. High costs and poor accessibility are other possible issues.

The aim of the present study was to assess the presence of DI using radiological features on CBCT.

## Materials and methods

Polish patients of both sexes were included in the study. Only high-quality scans were selected for analysis. Low-quality images, such as those with scattering or insufficient accuracy of bone borders, were excluded.

Consecutive CBCT scans taken from 2008 to 2016 were assessed. The examinations were performed using two devices: the Galileos (Sirona, Bensheim, Germany) (field of view [FOV], 15 cm in diameter; slice thickness, 0.15–0.30 mm) and the CS 9000 3D (Carestream Health, Rochester, NY, USA) (cylindrical FOV, 5 cm in diameter and 3.7 cm in height; slice thickness, 0.077 mm). Patients were referred due to various indications for CBCT scans. Apart from impacted teeth (7 patients) and supernumerary teeth (5 patients), the main reason for CBCT referral was assessment of the structure and position of the maxillary incisors (8 patients) (Table 1). Every CBCT scan in our clinic is performed according to a strict, standardized scanning protocol. All constructions and measurements in the present study were performed on a 21.3-inch flat-panel color-active matrix thin-film transistor medical display (Nio Color 3 M; Barco, Kortrijk, Belgium) with a resolution of 2048 × 1536 pixels at 76 Hz and 0.2115-mm dot pitch operated at 10 bits. All CBCT images were retrospectively evaluated by a radiologist (I.R.K.) with 20 years of experience and a dentist (M.P.) with 5 years of experience. Axial, sagittal, coronal, cross-sectional, and tangential images were reconstructed for all jaws, and three-dimensional reconstructions were used if necessary. The analysis focused on particular features of DI such as age, sex, side, lateralization, occurrence in a particular group of teeth, type of invagination, differentiation, and the consequences of their presence.

**Table 1:** Reasons for CBCT referral

Reason for CBCT referral	Patients (*n*)
Assessment of structure and position of a tooth	8
Impacted tooth	7
Supernumerary and impacted teeth	6
Supernumerary tooth	5
Trauma	1
Dens in dente	1
Teratoma	1
Malocclusion	1
Dilaceration	1
Transposition	1
Assessment of the buccal cortex of the maxilla	1
Total	33

*CBCT* cone-beam computed tomography

## Results

The study population comprised 33 patients [19 (57.6%) female, 14 (42.4%) male] who underwent CBCT imaging for dental abnormalities. The average age of the 33 patients was 15.48 years (standard deviation, 8.99 years; range 7–40 years). The mean age of the male patients was 9 years (range 8–38 years), while the mean age of the female patients was 12 years (range 7–40 years).

**Fig. 1:**
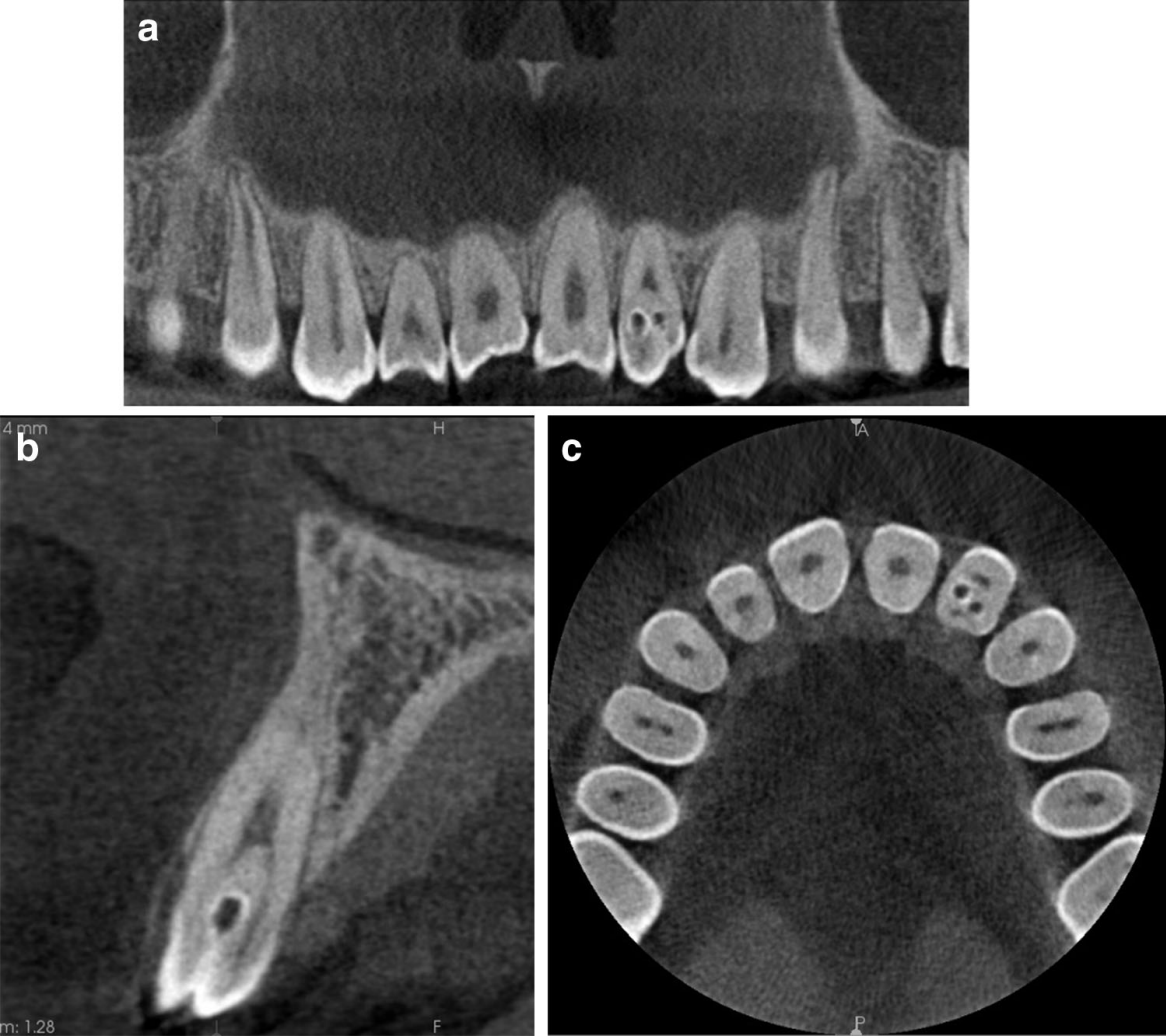
Example of coronal invagination of the left maxillary lateral incisor. Enamel in tooth 22 invaginated into the interior of the tooth, seen here as an opaque line. **a** Tangential view. **b** Cross-sectional image. **c** Axial plane

**Fig. 2:**
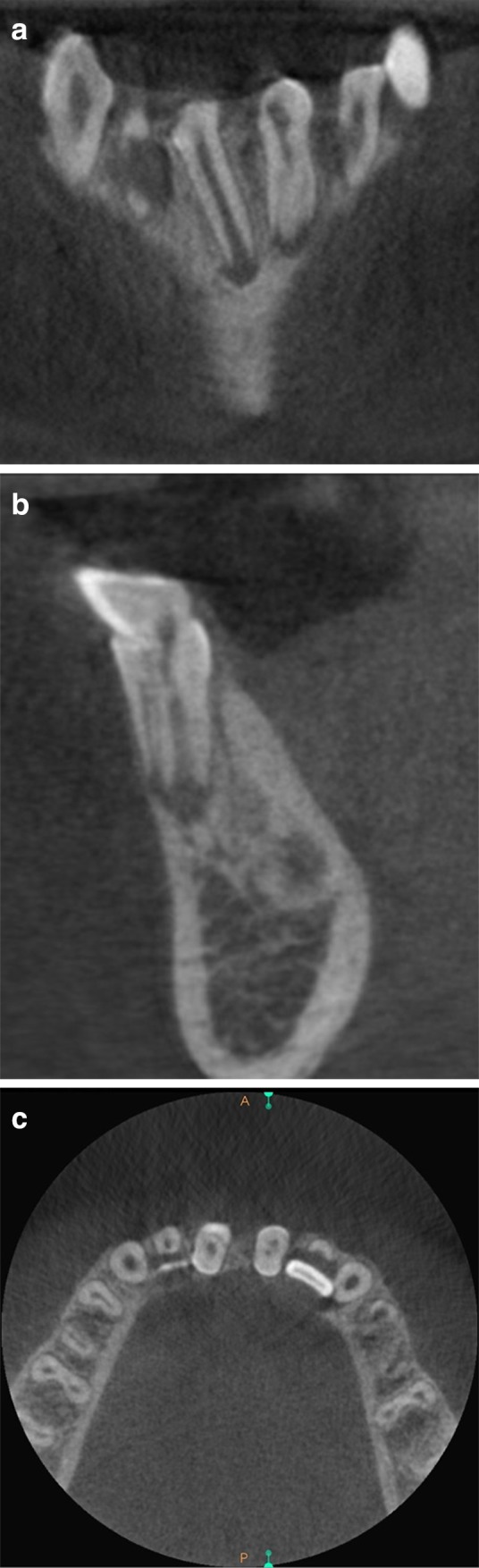
CBCT images of dens in dente anomaly of the left lower central incisor. **a** Tangential view. **b** Cross-sectional image. **c** Axial plane

**Fig. 3:**
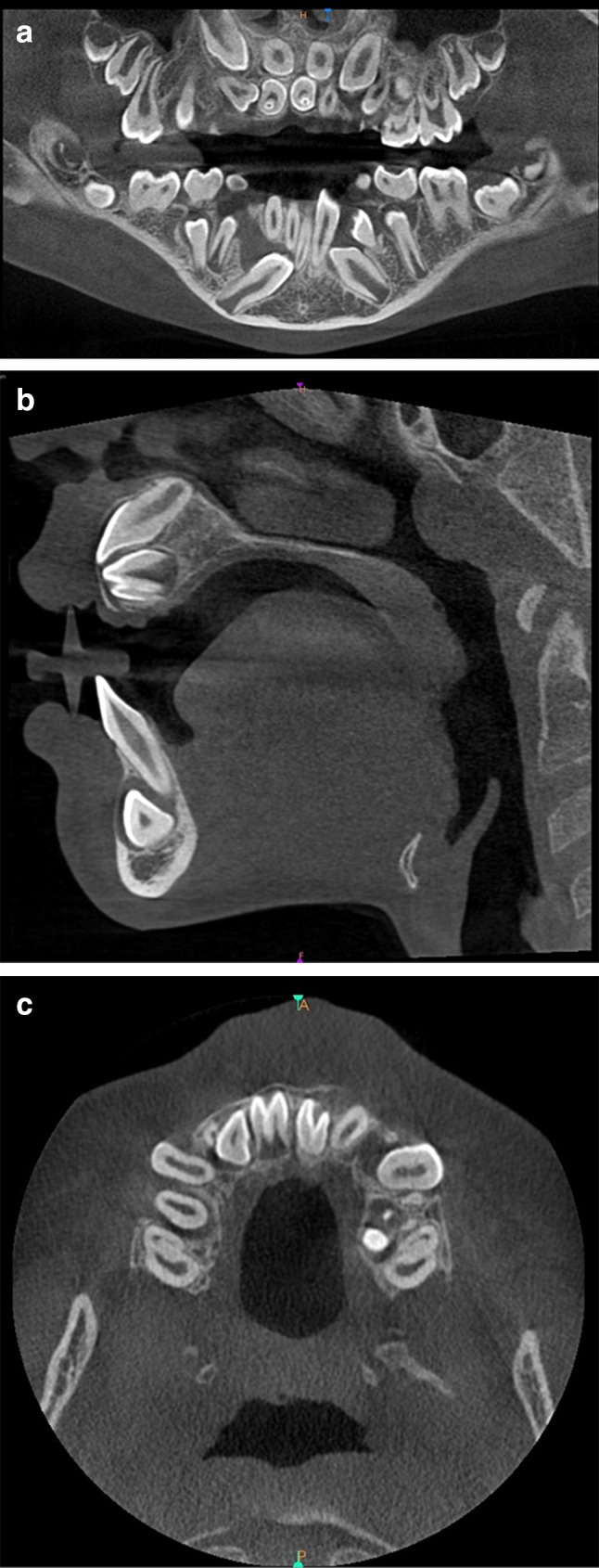
Example of bilateral occurrence of dens in dente of supernumerary, incorrectly developed and situated teeth 11′, 21′. **a** Panoramic view. **b** Cross-sectional image. **c** Axial plane

The invagination most frequently involved the maxillary lateral incisors [22 (53.7%) teeth], followed by supernumerary teeth (11′, 21′) [12 (29.3%) teeth] and the maxillary central incisors [2 (4.9%) teeth]. The invaginated teeth also included the maxillary second premolar, second molar, mandibular central incisor, and third molar. In total, 92.7% of the invaginated teeth were present in the maxilla, unilaterally rather than bilaterally (75.8 and 24.2%, respectively). Almost two-thirds (63.4%) of the affected teeth were found on the left side, and 36.6% were found on the right. The root type of DI was seen more frequently than the coronal type. According to Oehlers’ classification, the collected material comprised 6 type I teeth, 20 type II, 4 type IIIa, and 8 type IIIb. In some teeth with DI, it was impossible to distinguish types a and b because of incomplete root formation. Consequently, we classified three teeth as type III. The anatomy of the teeth with this anomaly changed in reference to both the crown and the root (Tables 2, 3; Figs. 1, 2, 3). The radiological features of DI are presented below.

**Table 2:** Occurrence of dens invaginatus in individual groups of teeth

Tooth	Maxilla	Mandible	Total
Molar	1	1	2
Premolar	1	0	1
Canine	0	0	0
Lateral incisor	22	0	22
Central incisor	2	1	3
Supernumerary	12	1	13
Total	38	3	41

**Table 3:** Occurrence of dens invaginatus depending on type of field of view, sex, age, tooth, and position in the dental arch

	FOV type	Sex	Age	Tooth	Side	FOV type	Sex	Age	Tooth	Side
1	Small	Female	7	21′	L	Small	Male	8	22	L
2	Small		7	31	L	Small		9	12, 22	R, L
3	Small		9	21	L	Small		9	11′, 21′	R, L
4	Small		10	22	L	Small		9	11′	R
5	Small		11	22, 12	R, L	Large		9	21	L
6	Small		11	11′	R	Small		10	11′, 21′	R, L
7	Small		11	22	L	Small		10	11′, 21′	R, L
8	Large		12	12, 22	R	Small		11	22	L
9	Large		13	21′	L	Small		13	12, 22	R, L
10	Small		13	22	L	Small		13	21′	L
11	Large		13	32′	L	Small		13	11′	R
12	Small		14	22	L	Small		33	12	R
13	Large		16	38	L	Large		33	12, 22	R, L
14	Small		18	22	L	Large		38	17	R
15	Large		19	12	R					
16	Large		19	22	L					
17	Large		22	12	R					
18	Large		28	12	R					
19	Large		40	25	L					

*FOV* field of view

### Characteristic features of invaginated crowns

In most cases, the crown had an irregular shape. Specifically, it was very wide (7.7–12.5 mm) compared with normal teeth from a particular group of teeth, and its length ranged from 4.7 to 20.0 mm. It also exhibited mesiodistal widening and vertical shortening and showed a peg-shaped or incorrect structure. The germ of the tooth with invagination developed in the coronal part only or developed improperly in the coronal part only.

### Characteristic features of roots with invagination

Roots with invagination were incorrectly formed with a wide or open apical foramen. The periapical area showed widening of the periodontal ligament (one tooth) and an osteolytic lesion of 9–16 mm in size with an ill-defined contour without the marginal ridge (six teeth).

### Radiological images of invagination

In the coronal type, the invagination extends from the palatal surface of the crown to the level of the cementoenamel junction. We found the presence of a pocket covered by enamel, discontinuity of the enamel layer, and an alternate system of opaque and translucent layers. In addition, we observed a surplus of hard tissues from the palatal side, whereas the enamel invaginated into the inner part of the tooth from the palatomesial surface.

In the root type, the invagination occurs within the crown and the root. We found the presence of tapered invagination of dentin in the central part of the root and a pocket covered by lamina dura in the apical region of the root.

The differentiation of DI according to type is presented in Table 4. The consequences were as follows:

**Table 4:** Differentiation of dens invaginatus depending of type of anomaly

Differentiation
Coronal type (type I)
Deep foramen cecum
Incorrectly formed, delayed tooth
Germ of mesiodens
Root type (types II, III)
Tooth germination with incomplete division
Fusion through the cementum of the regular tooth with the supernumerary tooth affected by dens invaginatus

1. Delayed resorption of the primary tooth root.
2. Delayed development compared with the corresponding contralateral tooth.
3. Delayed eruption compared with the corresponding contralateral tooth.
4. Impediment in eruption of a permanent tooth if the invagination involves a supernumerary tooth.
5. Pathological connection of the tooth with the oral cavity.
6. Rotation of the tooth crown and root deviation.
7. Displacement or deviation of adjacent teeth.

## Discussion

The development of radiodiagnostics has contributed to an increased treatment success rate for developmental malformations, meaning that patients can preserve their own dentition for a longer period of time. Because of the cause of DI was historically unknown and endodontic treatment could not be performed, affected teeth were simply extracted. CBCT examination with precise analysis of numerous thin slices has allowed for the performance of proper endodontic treatment even in very difficult cases [7]. Patel [8] claimed that it is impossible to assess the real nature of DI based on the conventional radiographs only; therefore, CBCT should be used when this anomaly is suspected. A periapical X-ray can clearly show the anomaly but does not depict its three-dimensional nature. It gives insufficient diagnostic information for the practitioner while assessing the true anatomy and planning the treatment. A case report by Patel [8] showed a particular advantage of CBCT. A periapical X-ray taken for diagnostic reasons revealed a lesion 4 mm in diameter associated with the mesial part of the root and signs of a class III invagination that appeared to have its own apical foramen. The periapical radiolucency appeared to be associated with the invagination. In this case, CBCT images confirmed the lesion but gave no information regarding its origin. The main canal was intact. The periapical lesion did not communicate with the main root canal; thus, only the invagination was treated. The root canal with the vital pulp was left untreated [8].

Depending on the source, the incidence of DI ranges from 0.04 to 10.00% in the general population [9] and from 1.30 to 12.00% in the Turkish population [10–12].

Few studies to date have focused on the prevalence of DI based on CBCT examination [13, 14]. Irrespective of the type of examination used during investigation, all studies (including the present study) have shown that the tooth most frequently affected by DI is the maxillary lateral incisor [13–15]. We found that 53.7% of all teeth affected by DI were maxillary lateral incisors. Decurcio et al. [14] discovered DI in 85.5% of cases and Capar et al. [15] in 75.0%, whereas Ceyhanli et al. [13] found DI in 86 (2.8%) of 3067 lateral incisors.

It is not rare to find DI in supernumerary teeth, particularly in the mesiodens [16, 17]. In the present study, 29.3% of all teeth with DI were supernumerary teeth. We encountered no patients with more than two DI-affected teeth (two teeth in 24.2% of patients and a single tooth in 75.8%), but some authors have reported patients with four DI-affected teeth (3.3% of patients), three teeth (2.2%), two teeth (29.7%), and one tooth (64.8%) [14].

Ceyhanli et al. [13] reported that 5.90% of all patients had at least one tooth with DI with no sex-related difference (5.31% of male patients and 6.57% of female patients). Likewise, no significant sex-related difference was found in the present study, and other studies seem to confirm this result [10, 12, 13, 18]. Only Gündüz et al. [11] reported a statistically higher prevalence of DI in female patients. In the present study, female patients were also more often affected, but the difference was not strongly emphasized. We also noticed that there was a stronger predilection for DI in the maxilla than in the mandible; 92.7% of DI-affected teeth were observed in the maxilla, and only 7.3% were found in the mandible. Surprisingly, the authors of some studies rarely found DI [14, 15, 18] or even found no DI in the mandible [13]. According to Oehlers’ classification, the most common type of DI is type I, depending on source (range 65.9–86.6% of cases) [4, 10, 13, 15]. In the present study, however, we found that type II was the most frequent type, as in the study by Decurcio et al. [14].

DI is more often found unilaterally, but bilateral occurrence is also possible. In the present study, DI was quite prevalent bilaterally (24.2% of cases), but other studies have revealed that bilateral occurrence is also frequent at 82.0% [12], 67.5% [11], 23.1% [10], and 15.6% of cases [13].

Many case reports have described successful management of DI diagnosed by means of CBCT [6, 7, 19–21]. In one case report, a tooth with type III DI with a large periapical lesion was treated simply by filling the invaginated canal. The main root canal was not affected, and, importantly, the vitality of the tooth was preserved [19]. Such treatment is possible only with a three-dimensional diagnosis.

CBCT allows for evaluation of the connection between the invagination and root canal, which is crucial when endodontic treatment is carried out to manage periapical lesions [6, 20].

CBCT allows for assessment of all necessary information regarding teeth with malformations such as DI, and this, in turn, facilitates the planning of treatment in all fields of dentistry. CBCT is particularly valuable in patients with type III DI for whom an endodontic treatment plan must be developed [6, 21, 22]. Using sagittal slices, the clinician can distinguish whether a periapical lesion arises from the main canal or from the invaginated one.

Knowledge of the presence of DI is crucial, because it affects the results of treatment in all fields of dentistry, particularly in endodontics. If DI is suspected, clinical examination is essential; careful radiographic interpretation is also required. To confirm the suspicion of DI, additional radiographic examination should be performed; CBCT is adequate for this purpose. CBCT with a small FOV is better than that with a large FOV, because the higher resolution exhibits more details. This particular examination is helpful in evaluating the type of anomaly. Many researchers have observed that DI occurs most frequently in the maxillary lateral incisors. Dental practitioners should be aware of the characteristic clinical features of DI and be able to recognize them using various radiological methods. They should also know which type of CBCT examination to choose to present the best view.

In conclusion, CBCT allows for precise diagnosis of DI. The most frequent location of this anomaly is the maxillary lateral incisor. CBCT allows the clinician to distinguish the type of abnormality, which is essential during treatment planning.
